# Groundwater Quality and Pollution Index for Heavy Metals in Saïs Plain, Morocco

**DOI:** 10.5696/2156-9614-10.26.200603

**Published:** 2020-05-04

**Authors:** Said Lotfi, Miloud Chakit, Driss Belghyti

**Affiliations:** 1 Laboratory of Agrophysiology, Biotechnology, Environment and Quality, Faculty of Sciences, Ibn Tofail University, Kenitra, Morocco; 2 Laboratory of Genetics, Neuroendocrinology and Biotechnology, Faculty of Sciences, Ibn Tofail University, Kenitra, Morocco

**Keywords:** groundwater quality, heavy metal, pollution index, Saïs plain

## Abstract

**Background.:**

Heavy metals contamination threatens groundwater resources in many areas around the world. Various methods to evaluate groundwater quality have been used to characterize sources of contamination and associated parameters. For assessment of heavy metals contamination, calculation of pollution indices is an effective tool for assessing water quality.

**Objectives.:**

The purpose of the present study was to assess heavy metal concentrations and determine distributions in Saïs plain, Morocco using multivariate analysis.

**Methods.:**

A total of 144 groundwater samples were collected from twelve stations in Saïs from January 2018 to January 2019, and were analyzed for heavy metals (arsenic, cadmium, total chromium, lead, copper, iron, manganese and zinc) using atomic absorption spectrophotometry.

**Results.:**

Chromium was found to be a major contaminant affecting water quality in Station 2 (0.057 mg/l) and Station 8 (0.065 mg/l), while elevated levels of iron were found in Station 7 (1.4 mg/l) and Station 11 (0.45 mg/l), and elevated levels of copper (2.9 mg/l) and zinc (3.39 mg/l) were found in Station 11, relative to other heavy metals. The high concentrations of these elements are related to anthropogenic pollutants. The factor analysis showed two components controlling groundwater chemistry. The results of the present study demonstrate that the concentrations of toxic metals, like Fe and Cr, are present in slight excess in one or two stations during one season. The calculated heavy metal pollution level for the groundwater of Saïs plain was below the index limit of 100.

**Conclusions.:**

The results show that groundwater is not polluted with respect to heavy metals and is acceptable for drinking. However, precautionary measures, such as managing the use of agricultural inputs and avoiding the use of wastewater in agriculture, are recommended in this area.

**Competing Interests.:**

The authors declare no completing financial interests

## Introduction

Groundwater resources are important sources of fresh water. Water quality is an important factor for ground and surface waters worldwide, and largely depends on a number of physicochemical parameters.^1^ Groundwater is used for domestic, agricultural and industrial purposes in most parts of the world. In addition to the increasing demands of a rapidly growing population, human activities such as agriculture and domestic usage release large amounts of pollutants into water bodies and influence water quality.^2^,^3^ Rivers and groundwater are used for domestic and agricultural purposes.^4^,^5^ Anthropogenic activities can deteriorate water quality. Contamination in urban areas is linked to human activities such as industrial and municipal discharges and leachate from landfill sites, while in rural areas, contamination is mainly connected to agricultural activities.^6^,^7^ These activities may include leachates from agricultural fields that use agro-chemicals as well as organic matter and drug residues from animal husbandry.^8^

Heavy metals contamination of groundwater sources has been reported across the globe.^9^–^11^ Although groundwater consumption is perceived to be safe, many studies have shown that groundwater may be susceptible to contamination. Anthropogenic activities, aquifer geology and climate are among the major factors that cause groundwater quality deterioration.^12^–^14^ Complex geological formations and flow domains are observed in groundwater resources. Therefore, hydrogeological and geochemical studies are essential in groundwater resource management and quality assessment. This is even more important when groundwater quality degradation is due to anthropogenic activities.^15^–^17^

Groundwater in Morocco is an important part of the water reserves and constitutes the main source of drinking water due to its ease of use and access.^18^ However, it is challenging to protect groundwater quality from pollution, especially rural areas. Groundwater pollution is a deeply concerning issue and the use of contaminated groundwater for consumption represents a health hazard.^19^

A study conducted by Darwesh *et al*. demonstrated that groundwater samples collected from the region of Sidi Slimane (Morocco) are considered unsuitable for drinking and groundwater quality is related to the geological structure of the area.^20^ Several studies have shown the impact of anthropogenic activities on groundwater quality.^18^,^21^ The objective of the present study was to evaluate spatial frequencies and distributions of heavy metal concentrations by applying multivariate statistical methods.

**Abbreviations**
*HPI*: Heavy metal pollution index
*PCA*: Principal component analysis

## Methods

**Figure 1:**
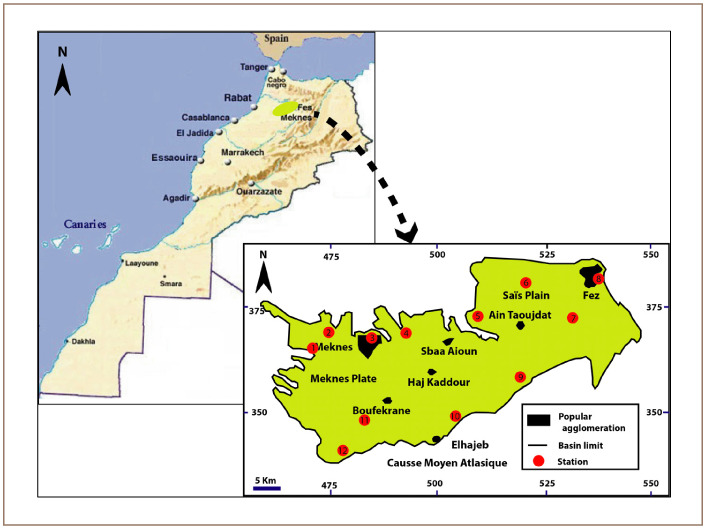
Map of study area and spatial distribution of 12 sampling stations

The study area is located in northern Morocco *(Figure 1).* The Saïs plain is one of Morocco's major agricultural zones. It contains two major cities, Fez in the east (more than1 million inhabitants),^22^ and Meknes in the west (679,996 inhabitants).^22^ Mean precipitation in the area is 500 mm/year, supporting numerous agricultural activities. The mean annual temperature is 17°C.

The climate of the Saïs region is semi-arid. It is characterized by irregular rainfall at spatial and temporal levels.^23^ According to data from the Sebou watershed, recorded during the period 1970–2003, the annual rainfall contributions vary between 643 mm per year in the west (Meknes) and 363 mm per year in the east (Fez).^24^Table 1 presents some characteristics of the twelve stations.

**Table 1:** Characteristics of Sampling Stations

**Stations**		**Well diameter (m)**	**Well depth (m)**	**Piezometric level (m)**	**Well status**	**Use of wells**	**Sources of pollution**
1	Ain Arma	1.45	28	20–26	Protected	Drinking water, domestic use and irrigation	Domestic wastewater, agricultural activities
2	Toulal	1.2	26	18–25	Not protected	Domestic use, and irrigation	Tannery, domestic wastewater
3	Wislane	1.5	25	16–24	Not protected	Drinking water, domestic use, and irrigation	Domestic and industrial wastewater, agricultural activities
4	SbaaAyo un	1.62	30	26–29	Protected	Drinking water, domestic use, and irrigation	Domestic wastewater and agricultural activities
5	Taoujdat	1.23	35	32–34	Not protected	Drinking water, domestic use, and irrigation	Domestic wastewater and agricultural activities
6	Ain Lahnach	1.53	27	19–25	Not protected	Drinking water, domestic use, and irrigation	Domestic wastewater and agricultural activities
7	Ain Chqaf	1.60	29	23–26	Not protected	Drinking water, domestic use, and irrigation	Domestic wastewater and agricultural activities
8	Sidi Hrazem	1.58	23	15–21	Not protected	Domestic use	Tannery, domestic wastewater and agricultural activities
9	BititAit Oualal	1.30	31	26–29	Not protected	Drinking water, domestic use, and irrigation	Domestic wastewater and agricultural activities
10	EL Hajeb	1.25	26	19–24	Not protected	Drinking water, domestic use, and irrigation	Domestic wastewater and agricultural activities
11	Mejjat	1.45	24	13–22	Not protected	Domestic use and irrigation	Industrial and domestic wastewater
12	Agourai	1.20	32	26–29.5	Not protected	Drinking water, domestic use, and irrigation	Domestic wastewater and agricultural activities

### Parameter analysis

A total of 144 groundwater samples were collected from twelve randomly selected stations in Saïs plain as outlined by Fifield and Haines.^25^ Collection bottles were rinsed several times with distilled water. Taps were allowed to run for at least 5 minutes prior to sample collection and labeled accordingly.

Samples were transported to the laboratory and then stored at −4°C for further analysis. The water samples were filtered on site and the concentrations of heavy metals (lead (Pb), cadmium (Cd), arsenic (As),manganese (Mn), iron (Fe), zinc (Zn), copper(Cu) and chromium (Cr)) were determined using an atomic absorption spectrophotometer with graphite furnace (VARIAN, 240, Zeeman) with a detection limit of 0.05 mg/l.^26^

### Index calculation

The overall quality of water can be examined using the heavy metal pollution index (HPI) method.^27^–^30^ The HPI is based on the weighted arithmetic quality mean method and was determined using Equation 1.^27^,^31^

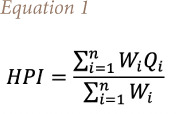
where, W_i_ is the unit weightage of i_th_ parameter, Q_i_ is the sub-index of i_th_ parameter and n is the number of considered parameters.

The sub-index of i_th_ parameter was computed using Equation 2.

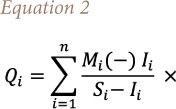
where, M_i_ is the monitored value of heavy metal of i_th_ parameter, I_i_ the ideal value of i_th_ parameter and S_i_ is the standard value of i_th_ parameter, in ppb. The negative sign (–) indicates the numerical difference of the two values, ignoring the algebraic sign. The critical value of HPI for drinking water is 100.^32^ The calculated index is intended for the analysis of drinking water.

The details of the calculations of HPI with unit weightage and standard permissible value as obtained in the study are given in Table 2. In order to calculate the HPI of water samples, the average concentration value of the selected metals (Cu, Fe, Zn, Mn, Cr, Pb, Cd, and As) was considered.^29^

**Table 2:** Standard Used for Index Computation^33^

**Parameter**	**MAC**	**Weightage**	**S**	**I**
Cu	1000	0.001	1000	2000
Fe	200	0.005	300	200
Zn	5000	0.0002	5000	3000
Mn	50	0.02	100	500
Cr	50	0.02	50	50
Pb	1.5	0.7	100	10
Cd	3	0.3	5	3
As	50	0.02	50	1

Abbreviations: MAC, Maximum admissible concentration/upper permissible; S, standard permissible in ppb; I, highest permissible in ppb.

### Statistical analysis

Factor and cluster analysis were used to evaluate the distribution of heavy metal components in different groundwater stations. Factor analysis was employed in order to reduce the number of variables and to analyze the relationship between these variables according to their common underlying factors using a reduced new set of orthogonal variables (principal components), arranged from the most to least important.

Principal component analysis (PCA) is a technique used to explain the variance of a large set of data containing variables that are inter-correlated, with a smaller set of independent variables.^34^ We employed PCA to compare the compositional patterns between the studied water systems and to extract the factors that influence each one of them.

Extraction of factors was done using varimax rotation and derived principal components with eigenvalue >1. The principal component method was used to study the distribution manner of individual association of elements in groundwater. Cluster analysis was employed to classify the heavy metals on the basis of their similarities within a group. Hierarchical agglomerative cluster analysis provides a similarity relationship between heavy elements using a dendrogram.

## Results

The present study assessed the concentration of heavy metals in groundwater samples collected from twelve stations located in the Saïs plain, Morocco. Heavy metals are naturally present in rocks and soils. However, the presence of metalliferous deposits contributes to metallic water contamination. The majority of contaminants come from industrial sources, such as mining or industrial activities. High concentrations of heavy metals can cause serious risks to humans, fauna and flora.

Concentrations of heavy metals varied at the water level of wells in the study area (Saïs plain). They were generally higher than the maximum allowable by the national standards for Fe, Cr, Cu and Zn.^35^ Concentrations were lower than the maximum allowable by national standards for As, Mn, Cd and Pb, which are known to be highly toxic to human health *(Table 3).*^35^

**Table 3:** Laboratory Analysis Results Compared to Allowable Limits

**Parameter**	**Minimum**	**Maximum**	**Mean**	**Moroccan standards^35^**	**World Health Organization^36^**
Pb	0	0.0029	0.00067	0.025	0.001
Cd	0	0.0019	0.00071	0.003	0.003
As	0	0.0019	0.00064	0.01	0.001
Mn	0	0.3	0.11	0.5	0.04
Fe	0	1.4	0.12	0.25	0.2
Zn	0	3.39	0.77	3	3
Cu	0	2.9	0.63	2	2
Cr	0	0.065	0.00382	0.05	0.05

Note: 144 samples, units in mg/l

### Lead

**Figure 2:**
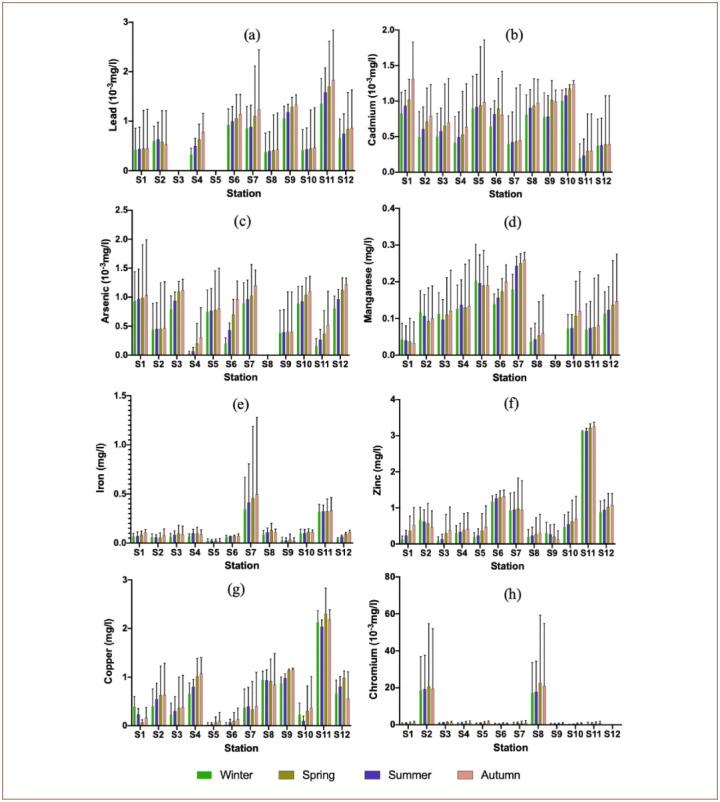
Seasonal variation of heavy metals in the 12 stations

Based on the results *(Figure 2a)*, the present study found that all Pb concentrations in the well waters of the Saïs basin were below the maximum allowable value (0.025 mg/l) of the Moroccan standard.^35^

### Cadmium

In the present study, all samples from the Saïs basin wells had values lower than the admissible Moroccan standards of 0.003 mg/l. No significant difference in Cd concentrations between seasons was observed in the study area (p <0.05%) (*Figure 2b)*. The maximum value found in all the samples of the Saïs basin was about 0.0019 mg/l.^37^

### Arsenic

According to the results of the analysis carried out during the sampling period, the As contents were found to be very weak and did not exceed 0.0019 mg/l. Therefore, they are all below the admissible Moroccan standards for drinking water (0.01 mg/l).^35^ According to seasons, no significant difference in As concentrations were observed in the study area (p <0.05%) *(Figure 2c).*

### Manganese

The Mn contents in the wells analyzed during the study period were much lower compared to the admissible Moroccan standards (0.5 mg/l) for drinking water. No significant difference in Mn concentrations was observed across seasons in the study area (p <0.05%) (Figure 2d).

### Iron

High concentrations of Fe were observed in Station 7 and Station 11 only throughout the study period with a slight seasonal fluctuation *(Figure 2e).*

### Zinc

Concentrations of Zn ranged from 0.00 to 3.39 mg/L with an average of 0.77 mg/l. One groundwater station (Station 11) had Zn levels slightly above the recommended value of 3 mg/l for drinking water.^36^ No significant difference in Zn concentrations was observed in the study area across seasons (p<0.05%) *(Figure 2f).*

### Copper

The average Cu concentrations observed in the study area showed lower levels compared to the maximum allowable value (2 mg/l) for most of the wells studied.^35^ High concentrations of Cu were observed in Station 11 throughout the study period with a slight seasonal fluctuation *(Figure 2g).*

### Chromium

The average total Cr concentrations observed in the study area were lower than to the admissible Moroccan standards (0.05 mg/l) for most of the wells studied. High concentrations of total Cr were observed in Station 2 (0.057 mg/l) and Station 8 (0.065 mg/l) throughout the study period with a slight seasonal fluctuation *(Figure 2h).*

### Heavy metal pollution index

The value 100 is the critical HPI, below which the overall pollution level is considered acceptable.^28^ As shown in Table 4, the HPI values obtained in the 12 stations were all under the maximum allowable HPI value in all seasons. The calculation of the index showed all the studied groundwater samples had an HPI under the critical pollution index value of 100 across all seasons.

**Table 4:** Heavy Metal Pollution Index of Water Samples

Station	S1	S2	S3	S4	S5	S6	S7	S8	S9	S10	S11	S12
HPI	33.25	38.35	39.4	40.25	34.79	35.97	41.21	34.90	34.51	31.77	43.14	41.97

### Factorial analysis

Factor analysis identified two factors responsible for 69.21% of the total variance in groundwater *(Table 5).*

**Table 5:** Total Variance Explained and Rotated Component Matrices for Heavy Metals

**Element**	**Component 1**	**Component 2**
As	−0.350	0.633
Cd	−0.160	−0.315
Cr	−0.007	−0.690
Cu	0.841	−0.218
Fe	0.567	0.190
Pb	0.801	0.048
Zn	0.646	0.340
Mn	−0.031	0.509
Explained variance	42.73	26.48

**Figure 3:**
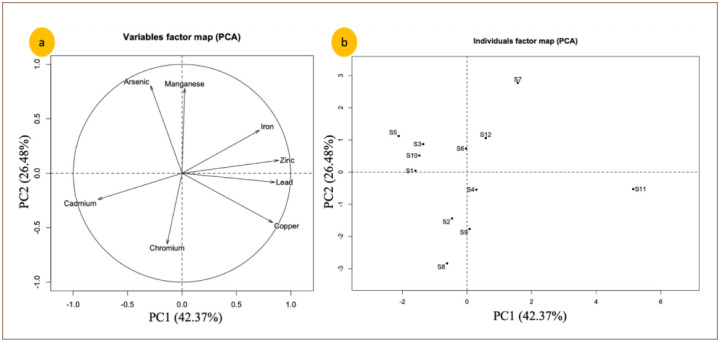
Plots of PCA (a) scores for distribution of water quality parameters in Saïs plain and (b) scores for combined data set of groundwater samples

Two components PC1 and PC2 of PCA showed 69.21% of the total variance of the obtained data of groundwater samples, as shown in Table 5. The first component (PC1) over 42.73% of the total variance in the dataset of groundwater; with Cu, Fe, Zn and Pb elements, indicating an industrial contamination. The second component (PC2) 26.48% of the total variance and showed strong positive loadings with Mn and As *(Figure 3a).*

Figure 3a shows the traits of samples and examines their spatial distribution. The samples that are located in the upper right quadrant are more enriched with Fe and Zn, while the lower right quadrant contains those that are enriched with Pb and Cu. The samples that are enriched with Cr, Cd and As (to a lesser extent) are distributed in the other two quadrants.

The scores plot (first component PC1 and second component PC2) for the groundwater samples *(Figure 3b)* shows high distribution of Cd and Cr elements in groundwater samples of Stations 2 and 8. These two samples are mainly distributed in the lower left quadrant, whereas the other sampling stations of Saïs basin are distributed in the upper left quadrant.

### Cluster analysis

**Figure 4:**
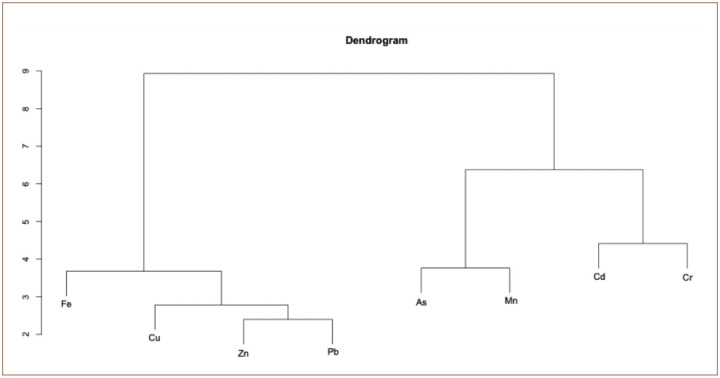
Dendrogram showing clustering of different heavy metals

The cluster analysis *(Figure 4)* resulted in two clusters of elements: the first one contained elements that had previously been interpreted as those derived from industrial activities (Fe, Cu, Zn and Pb), and the second cluster included elements derived from tannery activities (As, Mn, Cd and Cr).

## Discussion

### Lead

Lead is a toxic metal that can accumulate in the human body and over time causes damage to multiple vital organs. It is particularly harmful to fetuses, infants, and young children.^38^ In many parts of the world, its use has resulted in significant environmental contamination, human exposure and serious public health problems.^39^

Studies have found that chronic Pb exposure is linked to health problems such as anemia, hypertension in adults, kidney and brain damage in males, reproductive problems in women, and the formation of cataracts.^40^,^41^ The presence of low levels of Pb in water (less than 0.05 mg/l) can lead to nervous system damage, hearing problems as well as learning disabilities in infants, fetuses and young children.^42^

### Cadmium

Cadmium is a toxic element and has detrimental effects on aquatic life and human health.^43^ Several studies have shown that the consumption of very high amounts of Cd can cause lung and prostate cancer.^38^

### Arsenic

Drinking As-rich water over a prolonged period of time leads to As poisoning, known as arsenicism.^44^ Arsenicism can occur from an exposure time ranging from 5 to 20 years.^45^,^46^ Numerous studies have shown that long-term consumption of As-rich water leads to serious health effects. It causes non-cancerous dermal effects such as hyper- and hypo-pigmentation and keratosis, and increases the risk of diabetes, hypertension, and cardiovascular diseases.^47^,^48^

### Manganese

Manganese is a very common compound that is naturally occurring in air, soil, and water. Edmunds and Smedley and Edmunds *et al.* proposed that Mn can be produced incongruously or disproportionately during ionic reactions of silicate or oxide minerals.^49^,^50^ Manganese is naturally present in the environment, in solid form in soil and in the form of small particles in the human body. Particles of Mn in the air are present in the form of dust. They usually settle to earth within a few days. Industrial activity increases Mn concentrations in the air. Manganese produced from anthropogenic sources can adhere to surface water, groundwater, and sewage. Manganese enters the soil when Mn-rich pesticides are used. Although Mn is essential for the human body, it can be hazardous in high concentrations.^51^,^52^

### Iron

The presence of Fein water is linked to its abundance in the earth's crust.^53^,^54^ Mean Fe concentrations observed in the study area show levels below the admissible Moroccan standards (0.25 mg/l) for most of the wells studied.

The high values were likely due to industrial effects, as two wells of Station 11 were located near an industrial zone.

### Zinc

Zinc naturally exists in the air, water and soil, but can be unnaturally released in large quantities by anthropogenic activities and present human health hazards. Industrial activities, such as mining, coal burning and waste from the steel industry are the main sources of non-natural Zn.^55^ High values are likely due to industrial activities, as Station 11 is located near an industrial zone. According to Li and Zhang, the human body contains 2 to 3 g of Zn, around 90% is located in muscles and bones.^56^ Zinc is a vital nutrient and plays an indispensable role in maintaining human health by assisting in several aspects of cellular metabolism. However, long-term consumption of Zn in excess of the World Health Organization recommended values for drinking water can be hazardous to human health.^36^

### Copper

The presence of Cu in the environment comes from both natural and anthropogenic sources: mining, phosphate fertilizer production, paints and ceramics. High concentrations can cause health problems.^57^ The high values of Cu could be linked to industrial effects as Station 11 is located near an industrial zone.

### Total Chromium

Chromium exists in nature in different forms: Cr^+3^ and Cr^+6^. It can have several origins, either natural or anthropogenic, and has high environmental mobility.^58^,^59^ More than 70% of the total Cr in the environment comes from human activities such as non-ferrous smelters, refineries, tanneries, urban stormwater discharges, pulp and paper mill effluents and the discharges of thermal power stations. The toxicity of Cr in humans varies according to the form of the compound, its oxidation state and route of exposure. The high values could be due to industrial activities, as these two stations are located near a tannery.

The factor analysis indicates that high variations of Fe, Zn, Cu and Pb occurred in Station 11 due to industrial activities in this zone. Iron and Zn are associated in the first factor to Pb and Cu in the samples, which indicates that these metals may have the same source in the study zone. High concentrations of these metals were observed in zones located downstream of the city of Meknes. This indicates the contribution of anthropogenic sources. The presence of these metals might be due to industrial activities such as chemical industries and the papermill located in the industrial zone of the city of Meknes.^60^

The cluster analysis resulted in two clusters of elements: the first one contained elements that had previously been interpreted as those derived from industrial activities (Fe, Cu, Zn and Pb), and the second cluster included elements derived from tannery activities (As, Mn, Cd and Cr). The results show that ground water quality in the study area is threatened by pollutants and agrees with a study conducted in Sidi Slimane (Morocco).^20^ Except for Fe, the results of heavy metal evaluation in Saïs plain are similar to those found by El Baghdadi *et al*. in a study of groundwater in Beni Mellal city (Morocco).^61^

## Conclusions

Assessment of groundwater quality allows for the identification of significant parameters and thus obtains better information on contamination sources. The results of the present study suggest that presently, the concentration of metal ions is acceptable and does not reach levels that could be harmful to human health. However, the study clearly identifies concentrations of toxic metals such as Fe, Cr, etc., present in slight excess in one or two stations in a given season. Even though the current conditions meet existing standards, problems can occur in the future if conditions are not improved to prevent groundwater from becoming severely contaminated and unsafe for consumption. Appropriate preventative measures should be implemented to protect this important resource.

The HPI calculated for the groundwater of Saïs plain was found to be below the critical value of 100. This shows that groundwater in the study area is not polluted with respect to heavy metals. However, precautionary measures should be taken such as implementation of a groundwater quality monitoring program, preventing the use of wastewater in agriculture, controlling the overuse of organic fertilizers, monitoring the pre-treatment of wastewater (from factories) before discharge into the receiving environment and limiting the establishment of polluting industries.
